# Impact of the early reduction of cyclosporine on renal function in heart transplant patients: a French randomised controlled trial

**DOI:** 10.1186/1745-6215-13-231

**Published:** 2012-12-03

**Authors:** Pascale Boissonnat, Ségolène Gaillard, Catherine Mercier, Michel Redonnet, Bernard Lelong, Marie-Françoise Mattei, Annick Mouly-Bandini, Sabine Pattier, Agnès Sirinelli, Eric Epailly, Shaida Varnous, Marc-Alain Billes, Laurent Sebbag, René Ecochard, Catherine Cornu, François Gueyffier

**Affiliations:** 1 Hospices Civils de Lyon, Hôpital Louis Pradel, Pôle Médico-Chirurgical de Transplantation Cardiaque Adulte, 28, avenue du Doyen Lépine, F-69677, Bron Cedex, France; 2 INSERM, CIC 201, Lyon, Hospices Civils de Lyon, Service de Pharmacologie Clinique et Essais Thérapeutiques, Université Lyon 1, 7 Rue Guillaume Paradin, F-69000, Lyon, France; 3 Hospices Civils de Lyon, Service de Biostatistique, 162, avenue Lacassagne, F-69003, Lyon, France; 4 Département de Chirurgie Cardiaque, Hôpital Charles Nicolle, Université de Rouen, 1, rue de Germont, F-76000, Rouen, France; 5 Service de Cardiologie, Centre Hospitalier et Universitaire de Rennes, 2 rue Henri le Guilloux, F-35033, Rennes, France; 6 Département de Cardiologie et Transplantation, Hôpital Brabois, rue du Morvan, F-54511, Nancy, France; 7 Service de Chirurgie Cardiaque Adultes, Hôpital Timone, 264 rue Saint-Pierre, F-13385, Marseille, France; 8 Département de Cardiologie et Transplantation, Hôpital Guillaume et René Laënnec, boulevard Jacques Monod, F-44093, Nantes, France; 9 Service de Chirurgie Cardiaque, Centre Hospitalier Universitaire de Tours, 41, boulevard Béranger, F-37044, Tours, France; 10 Service de Chirurgie Cardiaque, Les Hôpitaux Universitaires de Strasbourg, 3, rue Koeberlé, F-67000, Strasbourg, France; 11 Département de Chirurgie Thoracique et Cardiovasculaire, Groupe Hospitalier la Pitié-Salpêtrière, 47-83 boulevard de l'hôpital, F-75013, Paris, France; 12 Département de Cardiologie et Transplantation, Centre Hospitalier du Haut Levèque, avenue de Magellan, 33604, Pessac, France; 13 CNRS and Université Lyon 1, UMR5558, Laboratoire de Biométrie et Biologie Evolutive, Equipe Biotatistique-Santé, 162, avenue Lacassagne, F-69003, Lyon, France; 14 CNRS and Université Lyon 1, UMR5558, Laboratoire de Biométrie et Biologie Evolutive, Equipe Modélisation et Evaluation des Thérapeutiques, 7 Rue Guillaume Paradin, F-69000, Lyon, France

**Keywords:** Calcineurin inhibition, Cyclosporine A, Heart transplantation, Renal function, Randomised clinical trial

## Abstract

**Background:**

Using reduced doses of Cyclosporine A immediately after heart transplantation in clinical trials may suggest benefits for renal function by reducing serum creatinine levels without a significant change in clinical endpoints. However, these trials were not sufficiently powered to prove clinical outcomes.

**Methods:**

In a prospective, multicentre, open-label, parallel-group controlled trial, 95 patients aged 18 to 65 years old, undergoing *de novo* heart transplantation were centrally randomised to receive either a low (130 < trough CsA concentrations <200 μg/L, n = 47) or a standard dose of Cyclosporine A (200 < trough CsA concentrations <300 μg/L, n = 48) for the three first post-transplant months along with mycophenolate mofetil and corticosteroids. Participants had a stable haemodynamic status, a serum creatinine level <250 μmol/L and the donors’ cold ischemia time was under six hours; multiorgan transplants were excluded. The change in serum creatinine level over 12 months was used as the main criterion for renal function. Intention-to-treat analysis was performed on the 95 randomised patients and a mixed generalised linear model of covariance was applied.

**Results:**

At 12 months, the mean (± SD) creatinine value was 120.7 μmol/L (± 35.8) in the low-dose group and 132.3 μmol/L (± 49.1) in the standard-dose group (*P* = 0.162). Post hoc analyses suggested that patients with higher creatinine levels at baseline benefited significantly from the lower Cyclosporine A target. The number of patients with at least one rejection episode was not significantly different but one patient in the low-dose group and six in the standard-dose group required dialysis.

**Conclusions:**

In patients with *de novo* cardiac transplantation, early Cyclosporine A dose reduction was not associated with renal benefit at 12 months. However, the strategy may benefit patients with high creatinine levels before transplantation.

**Trial registration:**

ClinicalTrials.gov NCT00159159

## Background

Cyclosporine A (CsA, calcineurin inhibitor) considerably improves survival after heart transplantation, leading to its widespread use over the last 25 years [1,2]. The last registry report of the International Society for Heart and Lung Transplant (ISHLT) provides overall survival rates of 80, 70, and 55% after one, five, and ten years, respectively [3].

This long-term survival is impaired by CsA side effects; in particular, nephrotoxicity that frequently leads to renal impairment. Retrospective studies have shown a 5 to 10% risk of chronic renal failure in patients treated with CsA after heart transplantation [2,4-6]. After transplantation of a non-renal organ, patients have a five-year risk of chronic renal failure from 7 to 21%, depending on what kind of organ is transplanted [5].

To limit renal impairment, several approaches have been explored in renal, cardiac, and liver transplantation. Immunosuppressive regimens have used mycophenolate mofetil (MMF) in combination with a calcineurin inhibitor (CNI) [7]. These efficient strategies foster the use of reduced CNI doses after the first high-risk rejection period after transplantation. In long-term heart transplant recipients with chronic renal dysfunction, a significant improvement in renal function and metabolic profile with no increased risk of acute rejection episodes was observed [8,9].

The use of reduced CsA doses immediately after renal transplantation has already been assessed in clinical trials [10-14]. Results suggest a benefit on renal function with a reduction in serum creatinine levels without a significant change in clinical endpoints (biopsy-proven rejection episodes, death). However, these trials were not sufficiently powered to prove clinical outcomes, but each of them adds a piece of the puzzle and should be available through publication in order to reduce the risk of bias when carrying out a systematic review.

Therefore, the reduction of CsA nephrotoxic effects using low doses immediately after heart transplantation, although attractive, has not been formally evaluated yet. The present study investigated the effect of an early low dose of CsA plus MMF on the change in serum creatinine level over a year in *de novo* heart transplant recipients.

## Methods

### Study design and ethics

This is a prospective, multicentre, open-label, randomised, parallel-group study. It was investigator designed. The study was approved by the Ethics Committee of the Centre Léon Bérard (Lyon, France), declared to the French Agency for the Safety of Health Products (AFSSAPS), registered on ClinicalTrials.gov under number NCT00159159, and conducted in accordance with the European Guidance for Good Clinical Practice and the Declaration of Helsinki.

### Randomisation and treatment

Within four days post-surgery, patients undergoing *de novo* heart transplantation were randomised to receive either a low dose or a standard dose of CsA, in a 1/1 ratio, as part of a triple immunosuppressant regimen, including MMF, and corticosteroids.

Patients were randomised using a centralised procedure. Randomisation was stratified by centre, age, presence of ischemia, and serum creatinine level before transplantation. Patients were followed up for 12 months. During the first three months, the low-dose group received CsA targeting a whole blood pre-dose concentration (C0) of 130 to 200 μmol/L, whereas the standard-dose group received CsA with a C0 between 200 and 300 μmol/L. Both doses reflect current practice [15]. Thereafter, to follow standard practice, the CsA doses of the standard-dose group were tapered to match the C0 of the low-dose group (130 μmol/L to 200 μmol/L).

The immunosuppressive treatment also included MMF (3 g daily) and corticosteroids according to local practice. MMF doses were adjusted according to individual tolerance.

All other concomitant antibacterial, antifungal, antiviral, anticholesterol, or antihypertensive drugs were administered at the discretion of physicians.

### Patients

Overall, 95 patients were enrolled in 10 French heart transplantation centres. Male and female heart transplant patients, aged 18 to 65, undergoing *de novo* heart transplantation were eligible. Donors’ cold ischemia time was under six hours. The main recipient exclusion criteria were: unstable haemodynamic status at the time of randomisation, circulatory assistance, serum creatinine level >250 μmol/L, multiorgan transplant, history of malignant disease within the past five years, a human immunodeficiency virus-positive blood test, a positive HB-antigen test, or a positive PCR hepatitis C test. Pregnant or breastfeeding women, patients participating in another trial, drug addicts, and psychiatric patients were also excluded. Each participant gave written informed consent.

### Study objectives, primary and secondary endpoints

The primary objective was to compare the renal function between the two groups by assessing changes in serum creatinine levels from inclusion up to 12 months. Creatinine clearance, microalbuminuria, and proteinuria were used as secondary endpoints. Immunosuppressive efficacy endpoints included the incidence of acute rejection episodes, the assessment of cardiac function (ejection and shortening fractions), and treatment failure (defined as death or withdrawal from the study for any reason). Any time during participation, the treatment was to be discontinued in patients of the low-dose group who received less than 1.5 g MMF/day for more than 15 consecutive days.

In compliance with the intention-to-treat (ITT) analysis of the primary endpoint, patients who withdrew early were followed up until the end of the study.

Strategy tolerance was assessed by the incidence of adverse events. Vital signs (weight and clinical manifestations) and cardiovascular risk factors (blood pressure, lipid, and glucose profiles) were closely monitored at each outpatient visit.

### Clinical follow-up

Following standard clinical procedures, the patients were monitored every two weeks during the first three months, then at months 4, 5, 6, 9, and 12 post-transplantation to assess the efficacy and safety of the strategy. Serum creatinine level was assessed at each visit. Creatinine clearance, proteinuria, and albuminuria were analysed at months 6 and 12. Cystatin C was assessed at inclusion, then at 1, 2, 3, 6, and 12 months.

Endomyocardial biopsies were performed according to local procedures. Biopsy-proven acute rejection episodes were graded by a local pathologist using the International Society for Heart and Lung Transplantation (ISHLT) scale. All biopsies performed over the study period were considered for analysis [16]. Acute rejection episodes were considered serious adverse events and the sponsor was immediately notified.

### Statistical analysis

Forty-five patients in each group (53 recruited for a drop-out of 15%) would ensure a 80% power to detect an 18% reduction in serum creatinine level of the low-dose group, at 12 months, assuming that the mean in the standard-dose group is 140 μmol/L, the common standard deviation is 43, and the two-sided two-group *t* test significance level is 0.05 [17].

According to the intention-to-treat (ITT) principle, analyses were performed on all 95 randomised patients.

Given biological variability and the imprecision of measurements, a mixed model of co-variance was used to take into account all available measures. The main analysis focused on a three- to twelve-month interval. It tested the effects of treatment at three months (low dose vs. standard dose) and whether the expected change lasted until the end of follow-up, when both groups started to receive the same low dose. Missing data were included as random events. The treatment effect was tested both on the intercept (treatment effect at three months) and on the slope (interaction between treatment and time of follow-up). The likelihood ratio test was used for this test comparing the models given below:

(1) $$Model\;1:\eta_{\mathrm{ij}}=\beta_{0}+\beta_{1}X1_{\mathrm{ij}}+U_{i0}$$

(2) $$Model\;2:\eta_{\mathrm{ij}}=\beta_{0}+\beta_{1}X1_{\mathrm{ij}}+\beta_{2}X2+\beta_{3}X1_{\mathrm{ij}}X2+U_{i0}+U_{i1}X1_{\mathrm{ij}}X2_{i}$$

Where η_ij_ is the predicted creatinine value (log10-transformed) for patient i and measurement j, X1_ij_ is the time elapsed after transplantation (in days), X2_i_ is the patient group, X1_ij_ X2_i_ is the interaction between the patient group and the time interval, U_i0_ is the random intercept for patient i, and U_i1_ is the random slope for patient i.

A box plot distribution was used to describe changes in creatinine level over time by treatment group.

A post hoc analysis was conducted on the primary endpoint dichotomised according to a clinically meaningful threshold (>120 μmol/L) in order to check that the low dose was able to decrease the proportion of patient over this threshold after three months and/or up to twelve months.

The threshold of 120 μmol/L was considered as clinically critical on the basis of literature data and clinical observations [18,19]. Our hypothesis was that the low dose could have a greater impact among the patients with the highest creatinine values. A hierarchical logistic regression model using a penalised quasi-likelihood method (glmmPQL) was used to test this hypothesis. The formula was similar to Model 2, but here, η_ij_ was the probability of having a creatinine level over the threshold (logit transformation).

The statistical analysis used R and SAS™ softwares.

## Results

**Figure 1:**
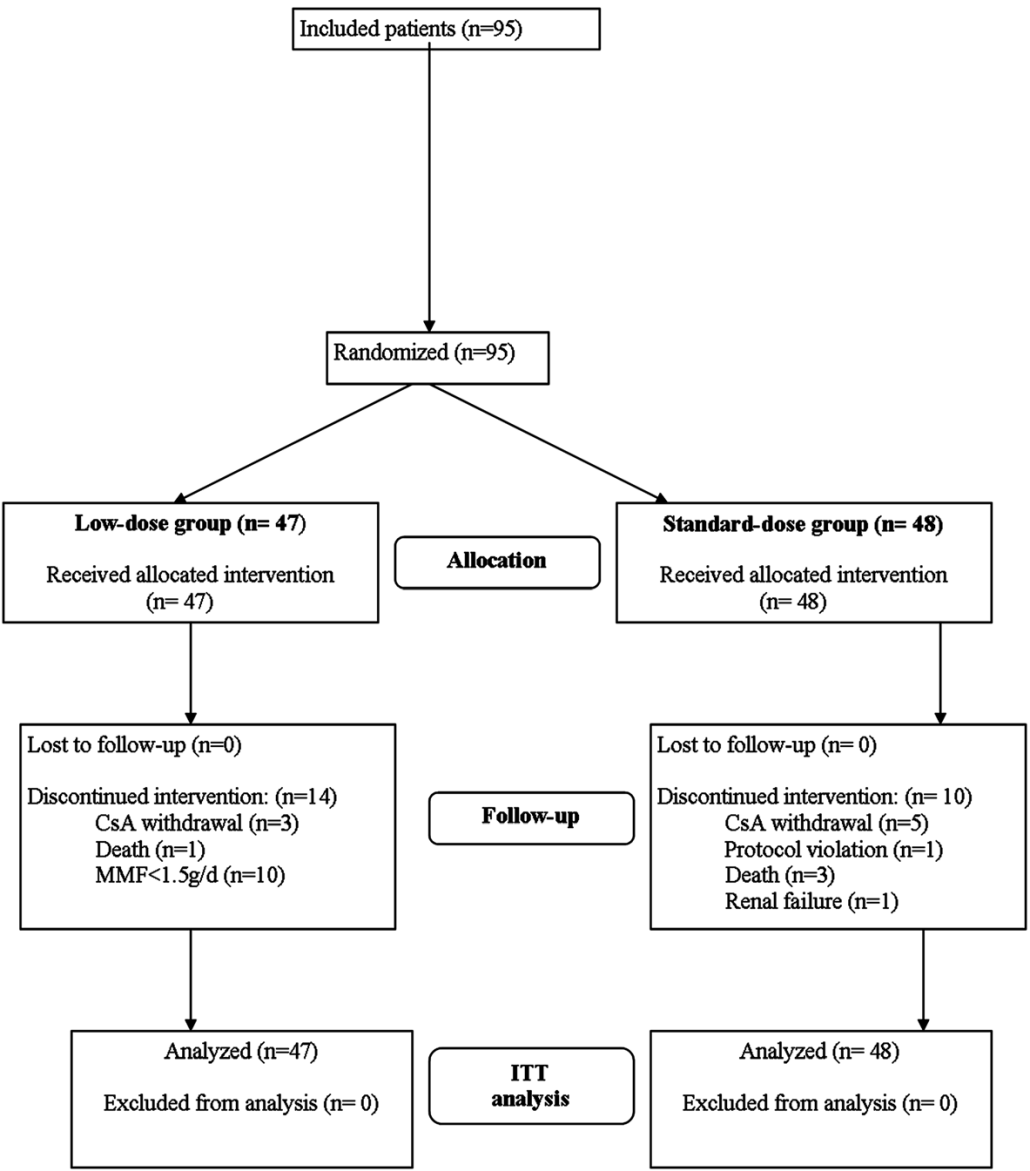
Study flow diagram

The flow diagram of the study is presented in Figure 1. Between March 2004 and December 2005, 95 eligible patients were included and randomised: 47 patients in the low-dose group and 48 in the standard-dose group of CsA treatment. The last follow-up visits took place in December 2006. All 95 patients underwent heart transplantation, received the assigned treatment, and were included in the ITT analysis.

The study was completed by 33 patients in the low-dose group and 38 patients in the standard-dose group. In the two groups, treatment discontinuation was the main reason for withdrawal; however, the number of discontinuations was lower in the standard-dose group than in the low-dose group (10.4% vs. 27.7%, *P* = 0.039).

The baseline characteristics of participants at inclusion are summarised in Table 1. Except for a higher proportion of females in the standard-dose group than in the low-dose group, all other variables were comparable in the two groups of patients.

**Table 1:** Baseline characteristics of the study population

**Characteristics**	**Low-dose group**	**Standard-dose group**
	**(n = 47)**	**(n = 48)**
Age, mean (SD), y	49.3 (10.2)	48.2 (11.5)
Sex		
Male n (%)	36 (76.6%)	29 (60.4%)
Female n (%)	11 (23.4%)	19 (39.6%)
Weight, mean (SD), kg	72 (14)	69 (16)
Serum creatinine, mean (SD), μmol/L	1247 (44.2)	126.5 (47.8)
Delay to cyclosporine A (CsA) use, mean (SD), d	1.9 (1)	1.9 (1)
Pre-transplantation on heart disease:		
Idiopathic, n (%)	18 (38.3%)	18 (37.5%)
Coronaropathy, n (%)	16 (34%)	18 (37.5%)
Congenital cardiopathy, n (%)	2 (4.3%)	3 (63.3%)
Valvular pathology, n (%)	1 (2.1%)	3 (63.3%)
Other, n (%)	10 (21.3%)	6 (12.5%)
Cytomegalovirus serology		
Donor^+^/Recipient^-^, n (%)		

C0 levels were generally met in the two groups with values in the upper end of the target range in the low-dose group. For the first three months, C0 levels were significantly higher in the standard-dose group than in the low-dose group (*P* <0.001) but the two group distributions overlapped with 16% of the values under the threshold of 200 μmol/L in the standard-dose group and 17% of the values over the threshold of 130 μmol/L in the low-dose group. As expected, from three months after heart transplantation until the end of the study, C0 levels were similar in the two groups. All patients received induction treatment with polyclonal antibodies except for one patient in the standard-dose group.

### Efficacy assessment

At 12 month post-transplantation, serum creatinine levels were available for 91 patients. At intermediate visits, data were available for 89 to 91 patients. Some data were missing for one to two patients at different study time-points. Observed creatinine values displayed strong individual variability. The results showed no significant difference in renal function changes between the two groups (*P* = 0.162) despite a trend towards a lower impairment of renal function in the low-dose group between three and twelve months (Table 2).

**Table 2:** Primary and secondary renal function outcomes

**Delay after heart transplantation**	**Low-dose group**		**Standard-dose group**		***P *value†**
	**n**	**Mean (SD)**	**n**	**Mean (SD)**	
Creatinine level					
At baseline	47	125(44)*	48	127(48)	0.59
Low versus standard cyclosporine A (CsA) regimen maintained:					---
At 0.5 month	47	101(30)	47	116(62)	---
At 1 month	47	105(40)	46	121(73)	---
At 1.5 month	47	106(44)	45	116(48)	---
At 2 months	47	104(42)	45	113(50)	---
At 2.5 months	45	101(40)	44	113(49)	---
At 3 months	46	103(30)	45	119(57)	0.062
Same CsA regimen:					
At 4 months	45	105(29)	44	123(46)	---
At 5 months	47	110(29)	44	126(47)	---
At 6 months	47	107(28)	45	128(72)	0.065
At 9 months	47	116(33)	45	131(49)	---
At 12 months	46	121(36)	45	132(49)	0.2
†*P* value of the global test (likelihood ratio test) for treatment effect after three months = 0.162					
Creatinine clearance					
6 months	27	76.7(31.4)**	33	66.1(29.4)**	NS
12 months	23	72.7(31.7)**	23	66.9(26.1)**	NS
Cystatin C					
3 months	36	1.21(0.44)††	33	1.5(0.7)††	0.04
6 months	35	1.25(0.34)††	31	1.5(0.9)††	NS
12 months	35	1.29(0.40)††	30	1.4(0.7)††	NS

*Creatinine values are expressed as means (SD) in μmol/L, **creatinine clearance values are expressed as means (SD) in mL/min, ††cystatin values are expressed as means (SD) in mg/L.

**Figure 2:**
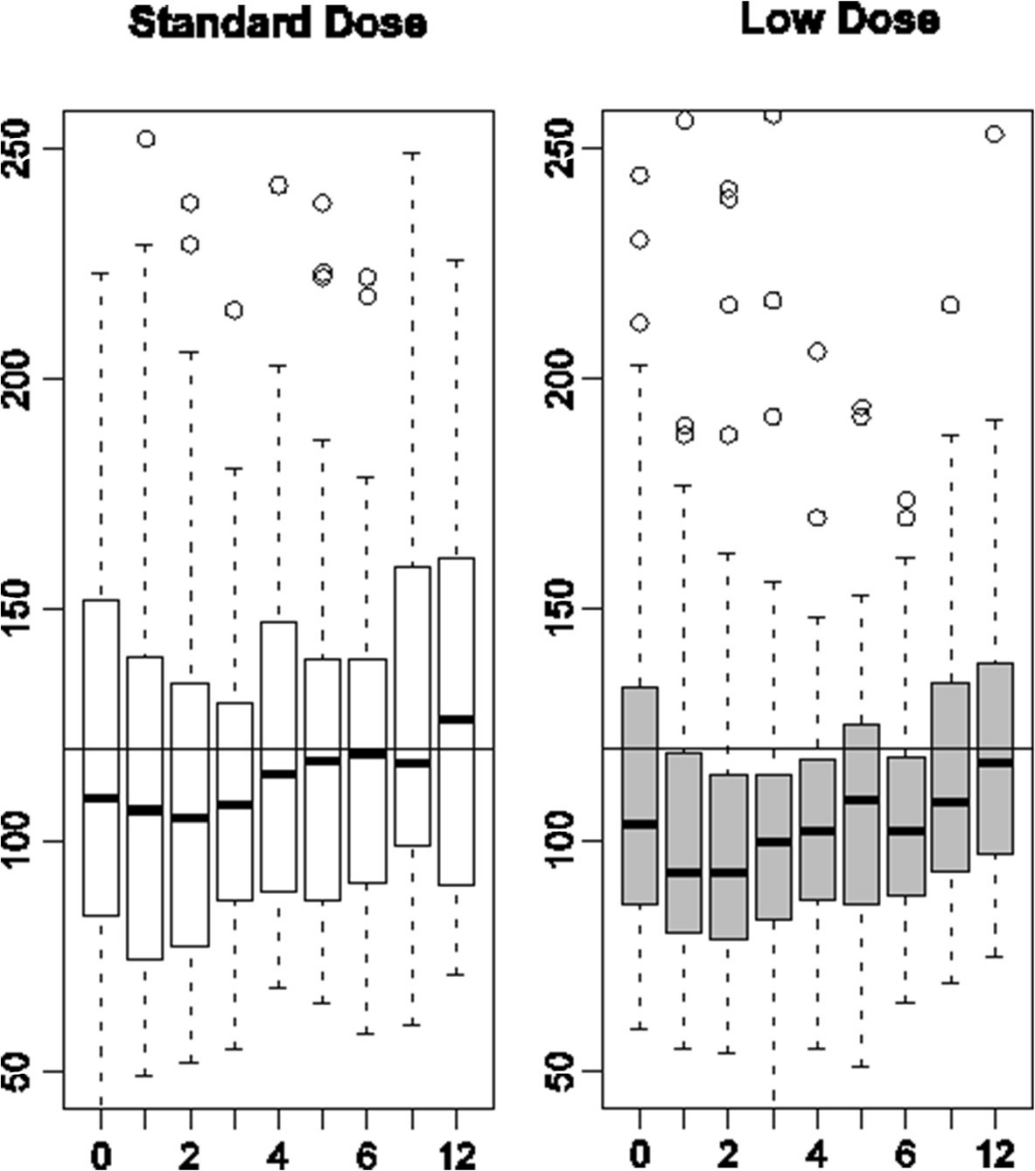
**Box plot distributions of observed serum creatinine values in standard-dose and low-dose patients by visit (months).** Reference horizontal line at 120 μmol/L.

Figure 2 presents a box plot distribution of observed serum creatinine values. After one month of treatment and during all of follow-up, the upper quartile was systematically lower in the low-dose group than in the standard-dose group. At three months, the post hoc analysis in patients with baseline creatinine >120 μmol/L showed a significantly lower number of patients above the clinically defined threshold in the low-dose group than in the standard-dose group (13% vs. 33%, *P* = 0.026) but the difference did not remain statistically significant thereafter and the treatment effect, as estimated by the hierarchical logical regression model on all available measures, was not statistically significant.

Other renal function parameters were favourable to the low-dose regimen despite the absence of significant differences (except for Cystatin C at three months) (Table 2).

### Safety assessment

#### Death

Four deaths were reported (Figure 1): one in the low-dose group (septic shock) and three in the standard-dose group (one herpetic infection with hepatocellular dysfunction, one pseudomonas septicaemia associated with cytomegalovirus infection, and one case of non-compliance with the immunosuppressive treatment). At 12 months, the survival was comparable between the low-dose (97.9%) and the standard-dose group (95.8%). No graft loss was reported during the study period.

#### Immunosuppressive efficacy

The incidence of acute rejection episodes, whatever their grade, was similar between the two groups (n = 41, 87.3%, in the low-dose group vs. n = 43, 91.5% in the standard-dose group). Treatment for rejection episodes graded 1B or higher concerned 20 patients (42.6%) in the low-dose group and 17 patients (36.2%) in the standard-dose group. The incidence of rejection episodes graded 3A or higher, usually taken as serious by clinicians, was 17% in the low-dose group and 14.6% in the standard-dose group (Table 3). The relative risk of severe acute rejection was 1.14 (95% confidence interval (0.45 to 2.90)).

**Table 3:** Histological grade of graft rejections

**Rejection grade**	**Low-dose group**		**Standard-dose group**		***P*value**
	**Patients with >1 episode**	**Assessment**	**Patients with >1 episode**	**Assessment**	
	**n (%)**		**n (%)**		
1A	38 (81%)	148	39 (83%)	178	NS
1B	20 (42.6%)	34	17 (36.2%)	45	NS
2	3 (6.4%)	6	8 (17%)	8	NS
3A	7 (14.9%)	9	7 (17.9%)	8	NS
3B	1 (2.1%)	1	0	0	NS

#### Study withdrawals

Treatment was discontinued in 10 patients of the low-dose group who received a MMF dose below 1.5 g/day for more than 15 consecutive days. Tacrolimus was used instead of cyclosporine in three patients of the low-dose group and five patients of the standard-dose group. Other reasons for withdrawal were one renal failure and one deviation from the protocol in the standard-dose group.

#### Other safety assessments

Excluding rejection episodes, at least one adverse event was reported in 91.6% of the participants: 93.6% in the low-dose group vs. 89.6% in the standard-dose group. Serious adverse events were reported in 69.5% of the participants: 72.3% in the low-dose group vs. 66.7% in the standard-dose group.

During the study period, more dialysis episodes were observed in the standard-dose group than in the low-dose group, but the difference was not statistically significant. Six patients had at least one dialysis episode: one (2.1%) in the low-dose group vs. five (10.4%) in the standard-dose group (*P* = 0.097).

The overall incidence of infections was similar between both groups. The incidence of cytomegalovirus infection (PCR-positive, and/or infection) was 30% in the low-dose group and 25% in the standard-dose group.

At 12 months, the mean systolic and diastolic blood pressures were not significantly different: 136 ± 16.1 vs. 134 ± 13.5 and 85 ± 14 vs. 85 ± 11 in the low-dose and standard dose groups, respectively. All patients received statins and mean total cholesterol and triglyceride levels remained within normal ranges and did not differ between the two groups (5.09 ± 1.12 vs. 5.07 ± 1.01 mmol/L for cholesterol and 1.66 ± 0.86 vs. 1.58 ± 0.84 mmol/L for triglycerides in the low-dose and the standard-dose groups, respectively).

## Discussion

Over 12 months, we observed less renal function impairment in heart transplant patients receiving a low dose of CsA than in those receiving a standard dose. However, differences in creatinine levels between three and twelve months were not statistically significant.

Several reasons may explain these results. First, the observed difference at 12 months (12 μmol/L) was lower than expected (24 μmol/L for an 18% reduction). Second, during the period under different dose regimens, the two group distributions of blood CsA values overlapped. During the first three months in the low-dose group, the C0 level remained at the upper end of the target range reflecting difficulties for physicians to significantly reduce CsA doses. This difficulty has already been reported [9]. Third, the primary endpoint was assessed at 12 months; that is, after nine months of identical dose regimens but the observed difference decreased after three months under the same regimen. Therefore, the low-dose strategy was not long enough to have a strong impact on the results at 12 months. Fourth, the important heterogeneity of individual responses over time has probably affected the power of the analysis. Finally, the lower frequency of high creatinine values (upper quartile) between three and twelve months in the low-dose group suggests different response profiles possibly linked to the severity of renal impairment. Even though less patients with severe renal impairment (creatinine >120 μmol/L) were seen at three months in the low-dose group, the post hoc analysis was not able to show a statistically significant effect, possibly because of a lack of power.

Furthermore, a lower number of dialysis episodes were reported in the low-dose group than in the standard-dose group, but this was not statistically significant. A meta-analysis by subgroup is being conducted to confirm whether or not patients with severe renal impairment benefit from low-dose cyclosporine after heart transplant. Other renal function indicators (creatinine clearance, cystatin C) progressed at the same rate as our primary endpoint, which is clinically consistent.

Overall, cardiac function remained stable and comparable between the two treatment groups and, consistently with other heart-transplant studies, serious adverse events were frequent but not different between the two groups.

An important number of patients under low-dose CsA withdrew from the study because of intolerance to MMF. In the study protocol, MMF <1.5 g/day for more than 15 consecutive days was a reason for withdrawal only in the low-dose group. Also, at the time of the trial, the choice was made to maintain patients at 3 g MMF/day without controlling blood levels. However, in patients with renal insufficiency recommendations were changed to a reduction of MMF dosage to 2 g/day and the use of blood-level monitoring [20].

The present trial showed that there was no difference between the two groups regarding the incidence of severe acute rejection episodes. However, due to the small size of the study, an increased risk of severe acute rejection episodes in the low-dose group cannot be excluded. The use of post hoc combined criteria that include dialysis plus death (two cases in the low-dose group vs. eight cases in the standard-dose group, *P* = 0.049) may lead to benefits when reducing CsA soon after heart transplantation. These results could be relevant to 25% of patients in the first year after heart transplant, who take CsA as a first-line treatment [21].

Several limitations of our study have to be acknowledged. These include the small number of new heart transplantations in France, possible biases related to the open-label design of the study, the choice of an endpoint devoid of direct clinical relevance, and the insufficient power to address all dimensions of the risk/benefit ratio of the newly proposed strategy. Nevertheless, this study is the first French multicentre trial in heart transplantation that addressed this daily specialist concern. Obviously, our results cannot be viewed as a definitive answer to the issue of the risk/benefit ratio of the CsA reduction strategy, but they may foster future research in the same direction and orient future studies towards a success/failure approach. They explore whether patients fitting particular individual profiles are more likely to benefit from a low-dose CsA strategy.

## Conclusions

In patients with *de novo* cardiac transplantation, early Cyclosporine A dose reduction was not associated with renal benefit at 12 months. However, the strategy may benefit patients with high creatinine levels before transplantation.

## Competing interests

This work was supported by Hospices Civils de Lyon and Roche. The authors declare they have no competing interests.

## Authors’ contributions

Here is a summary of the level of participation of each author: BP (main project coordinator, article author, patient recruiter), GS (co-author, project coordinator), MC (statistical analysis), RM (recruited largest number of patients), LB (second recruiter), MMF (third recruiter), M-BA (fourth recruiter), PS (fifth recruiter), EE (sixth recruiter), VS (seventh recruiter), BMA (eighth recruiter), SL (recruiter at the coordinating centre), ER (statistical analysis), CC (interpretation of results), GF (methodology). All authors read and approved the final manuscript.

## References

[B1] Hosenpud JD Bennett LE Keck BM Fiol B Boucek MM Novick RJ The registry of the international society for heart and lung transplantation: fifteenth official report–1998 J Heart Lung Transplant 1998 17 656 668 9703230

[B2] Fritsche Impact of cyclosporine on the development of immunosuppressive therapy Transplant Proc 2003 Suppl 2S 130S 134S 10.1016/j.transproceed.2003.12.038 15041322

[B3] Taylor DO Stehlik J Edwards LB Aurora P Christie JD Dobbels F Kirk R Kucheryavaya AY Rahmel AO Hertz MI Registry of the international society for heart and lung transplantation: twenty-sixth official adult heart transplant report-2009 J Heart Lung Transplant 2009 28 1007 1022 10.1016/j.healun.2009.08.014 19782283

[B4] Goldstein DJ Zuech N Sehgal V Weinberg AD Drusin R Cohen D Cyclosporine-associated end-stage nephropathy after cardiac transplantation: incidence and progression Transplantation 1997 63 664 668 10.1097/00007890-199703150-00009 9075835

[B5] Ojo AO Held PJ Port FK Wolfe RA Leichtman AB Young EW Arndorfer J Christensen L Merion RM Chronic renal failure after transplantation of a nonrenal organ N Engl J Med 2003 349 931 940 10.1056/NEJMoa021744 12954741

[B6] Pattison JM Petersen J Kuo P Valantine V Robbins RC Theodore J The incidence of renal failure in one hundred consecutive heart-lung transplant recipients Am J Kidney Dis 1995 26 643 648 10.1016/0272-6386(95)90602-9 7573020

[B7] Kobashigawa J Miller L Renlund D Mentzer R Alderman E Bourge R Costanzo M Eisen H Dureau G Ratkovec R Hummel M Ipe D Johnson J Keogh A Mamelok R Mancini D Smart F Valantine H A randomized active-controlled trial of mycophenolate mofetil in heart transplant recipients. Mycophenolate Mofetil Investigators Transplantation 1998 66 507 515 10.1097/00007890-199808270-00016 9734496

[B8] Ekberg H Bernasconi C Tedesco-Silva H Vítko S Hugo C Demirbas A Acevedo RR Grinyó J Frei U Vanrenterghem Y Daloze P Halloran P Calcineurin inhibitor minimization in the Symphony study: observational results 3years after transplantation Am J Transplant 2009 9 1876 1885 10.1111/j.1600-6143.2009.02726.x 19563339

[B9] Gaston RS Kaplan B Shah T Cibrik D Shaw LM Angelis M Mulgaonkar S Meier-Kriesche HU Patel D Bloom RD Fixed- or controlled-dose mycophenolate mofetil with standard- or reduced-dose calcineurin inhibitors: the Opticept trial Am J Transplant 2009 9 1607 1619 10.1111/j.1600-6143.2009.02668.x 19459794

[B10] Cohen DJ Vincenti F A comparative open label study to evaluate graft function in de novo renal allograft recipients treated with reduced dose or standard dose cyclosporine in combination with sirolimus and corticosteroids Am J Transplant 2003 Suppl 5 S465

[B11] de Sévaux RG Gregoor PJ Hené RJ Hoitsma AJ Vos P Weimar W Van Gelder T Hilbrands LB A controlled trial comparing two doses of cyclosporine in conjunction with mycophenolate mofetil and corticosteroids J Am Soc Nephrol 2001 12 1750 1757 11461949 10.1681/ASN.V1281750

[B12] Gonwa TA Hricik DE Brinker K Grinyo JM Schena FP Sirolimus Renal Function Study Group Improved renal function in sirolimus-treated renal transplant patients after early cyclosporine elimination Transplantation 2002 74 1560 1567 10.1097/00007890-200212150-00013 12490789

[B13] Kahan BD Julian BA Pescovitz MD Vanrenterghem Y Neylan J Sirolimus reduces the incidence of acute rejection episodes despite lower cyclosporine doses in caucasian recipients of mismatched primary renal allografts: a phase II trial. Rapamune Study Group Transplantation 1999 68 1526 1532 10.1097/00007890-199911270-00016 10589950

[B14] Muhlbacher F Paczek L An open-label study to evaluate the efficacy and safety of cyclosporine reductionin de novo renal allograft recipients receiving sirolimus: a dose comparative study Am J Transplant 2002 Supp 3 S238

[B15] Kobashigawa JA Miller LW Russell SD Ewald GA Zucker MJ Goldberg LR Eisen HJ Salm K Tolzman D Gao J Fitzsimmons W First R Tacrolimus with mycophenolate mofetil (MMF) or sirolimus vs. cyclosporine with MMF in cardiac transplant patients: 1-year report Am J Transplant 2006 6 1377 1386 10.1111/j.1600-6143.2006.01290.x 16686761

[B16] Stewart S Winters GL Fishbein MC Tazelaar HD Kobashigawa J Abrams J Andersen CB Angelini A Berry GJ Burke MM Demetris AJ Hammond E Itescu S Marboe CC McManus B Reed EF Reinsmoen NL Rodriguez ER Rose AG Rose M Suciu-Focia N Zeevi A Billingham ME Revision of the 1990 working formulation for the standardization of nomenclature in the diagnosis of heart rejection J Heart Lung Transplant 2005 24 1710 1720 10.1016/j.healun.2005.03.019 16297770

[B17] Ekberg H The use of daclizumab and mycophenolate mofetil in combination with corticosteroids and cyclosporing (low dose versus low dose followed by withdrawal) to optimize renal function in recipients of renal allografts [poster presentation] 2004 International Conference of the Transplantation Society (ICTS), Vienna, Austria

[B18] Agence Nationale d'Accréditation et d'Evaluation en Santé [Diagnosis of adult chronic kidney failure] Diabetes Metab 2003 29 315 324 French 12909822 10.1016/s1262-3636(07)70043-5

[B19] Couchoud C Pozet N Labeeuw M Pouteil-Noble C Screening early renal failure: cut-off values for serum creatinine as an indicator of renal impairment Kidney Int 1999 55 1878 1884 10.1046/j.1523-1755.1999.00411.x 10231450

[B20] van Gelder T Mycophenolate blood level monitoring: recent progress Am J Transplant 2009 9 1495 1499 10.1111/j.1600-6143.2009.02678.x 19519824

[B21] Hertz MI Aurora P Benden C Christie JD Dobbels F Edwards LB Kirk R Kucheryavaya AY Rahmel AO Rowe AW Stehlik J Scientific registry of the international society for heart and lung transplantation: introduction to the 2011 annual reports J Heart Lung Transplant 2011 30 1071 1132 10.1016/j.healun.2011.08.002 21962015

